# Exploring the Association of Vitamin D and VDR Polymorphisms with Disease Severity in COVID-19 and Influenza

**DOI:** 10.3390/biomedicines13112617

**Published:** 2025-10-25

**Authors:** Alexandru Constantin Sîrbu, Ioana Corina Bocșan, Octavia Sabin, Raluca Maria Pop, Ștefan Cristian Vesa, Gavriela Feketea, Violeta Briciu, Mihaela Lupșe, Anca Dana Buzoianu

**Affiliations:** 1 Department of Pharmacology, Toxicology and Clinical Pharmacology, Iuliu Hațieganu University of Medicine and Pharmacy, 400337 Cluj-Napoca, Romania; alexandru.sirbu@umfcluj.ro (A.C.S.); octavia.sabin@umfcluj.ro (O.S.); raluca.pop@umfcluj.ro (R.M.P.); stefan.vesa@umfcluj.ro (Ș.C.V.); abuzoianu@umfcluj.ro (A.D.B.); 2 Pediatric Allergy Outpatient Clinic, Department of Pediatrics, “Karamandaneio” Children’s Hospital of Patra, 26331 Patras, Greece; g.feketea@karamandaneio.gov.gr; 3 Department of Infectious Diseases and Epidemiology, “Iuliu Hatieganu” University of Medicine and Pharmacy, 400348 Cluj-Napoca, Romania; briciu.tincuta@umfcluj.ro (V.B.); mihaela.lupse@yahoo.com (M.L.); 4 Clinical Hospital of Infectious Diseases, 400348 Cluj-Napoca, Romania

**Keywords:** vitamin D, COVID-19, influenza, respiratory infections, vitamin D receptor (VDR), polymorphisms, SNP, disease severity, SARS-CoV-2

## Abstract

**Background**: Respiratory viral infections such as COVID-19 and influenza represent significant public health threats, especially in vulnerable populations. Vitamin D has been promoted as an immunomodulatory agent, with previous studies suggesting an association between vitamin D levels and disease severity in respiratory infections. Additionally, genetic variations in the vitamin D receptor (VDR) may influence immune responses. **Methods**: This study investigates the relationship between vitamin D levels, VDR polymorphisms (rs1544410, rs731236, and rs7975232), and clinical severity in hospitalized patients with COVID-19 and influenza during the 2023–2024 winter season. **Results**: A total of 71 patients were included in this cross-sectional study. Vitamin D levels were significantly lower in severe COVID-19 cases (8.08 ng/mL, IQR: 4.79–15.7) compared to moderate forms (32.6 ng/mL, IQR: 13.0–38.6), as well as severe influenza cases (25.6 ng/mL, IQR: 18.9–34.5). Additionally, severe COVID-19 patients exhibited higher inflammatory markers (CRP, neutrophil count) and lower lymphocyte counts. However, no significant association was found between VDR polymorphisms and disease severity or vitamin D levels. **Conclusions**: These findings highlight the potential role of vitamin D in modulating disease severity in respiratory viral infections, while the influence of genetic polymorphisms remains uncertain. Further research is needed to determine whether vitamin D supplementation could improve clinical outcomes in these infections.

## 1. Introduction

Respiratory viral infections represent a significant public health burden, especially during the winter season, when the circulation of highly transmissible viruses, such as SARS-CoV-2 and influenza, peaks. The 2023–2024 winter season was marked by the co-circulation of various SARS-CoV-2 variants, alongside seasonal influenza [1,2,3]. These infections can range in severity from mild respiratory symptoms to severe complications, including pneumonia, acute respiratory distress syndrome (ARDS), and even death, particularly in vulnerable populations such as the elderly and individuals with chronic comorbidities. Considering the risks that respiratory viruses pose, there is an ongoing search for treatments and preventable risk factors that can alleviate the disease burden and improve the clinical outcome [4,5,6]. Among these, the role of vitamin D in immune regulation has gained increasing attention.

Vitamin D has immunomodulatory properties [7,8] and vitamin D supplementation has been proposed as an overall protective agent against acute viral infections [9]. Several studies have explored the relationship between vitamin D levels and susceptibility to specific respiratory infections, including influenza and COVID-19. A meta-analysis suggests that vitamin D supplementation has a preventive effect on influenza [10]; however, some authors consider that more data is needed to confirm its role [11].

In patients suffering from COVID-19, lower vitamin D levels were associated with a higher infection rate, increased severity of disease, and mortality [12]. Evidence for the use of vitamin D supplementation in treating patients infected with SARS-CoV-2 is uncertain [13], but it leans towards a potential use in reducing hospital stay duration and ICU admission rates [14].

In addition to serum vitamin D levels, genetic factors such as polymorphisms in the vitamin D receptor (VDR) gene may influence vitamin D metabolism and the immune response to respiratory viral infections [15,16]. A meta-analysis shows that certain VDR gene polymorphisms can influence the response to vitamin D supplementation [17]. Some of the most studied polymorphisms of VDR are BsmI (rs1544410), TaqI (rs731236), and ApaI (rs7975232). The BsmI polymorphism is located in intron 8 and reflects a T(A) to C(G) change, with the T sometimes designated as the B allele and the C as the b allele [18]. It has been studied for immunity-related conditions, such as asthma [19], multiple sclerosis [20], and autoimmune thyroid disease [21], and has a possible role in IFN-gamma modulation [22]. TaqI is located in exon 9 of the VDR gene and reflects a synonymous substitution with an A(T) to G(C) change. Previous studies suggest that it may be involved in type I diabetes prevalence [23] and it may also present an association with systemic lupus erythematosus risk [24]. Finally, the ApaI polymorphism, which is also located in intron 8, can be presented as either C(G) or A(T) alleles. It has been investigated for its potential impact on immune function and potential susceptibility to the development of myasthenia gravis [25], systemic lupus erythematosus [26], and Behcet’s disease [27].

Understanding the interplay between these genetic factors, vitamin D levels, and the immune response could provide insights into the heterogeneity of clinical responses to respiratory infections. However, only limited studies have systematically investigated these relationships in the context of severe influenza and COVID-19 [28,29].

This study aims to address these gaps by evaluating vitamin D levels and VDR polymorphisms and their association with clinical and biological outcomes in patients hospitalized with severe influenza and COVID-19 during the 2023–2024 winter season. By integrating clinical, biochemical, and genetic data, this research aims to explore the potential role of vitamin D metabolism and genetic variations in shaping the severity and progression of respiratory viral infections.

## 2. Materials and Methods

### 2.1. Study Design and Setting

We conducted a cross-sectional observational study at the Clinical Hospital for Infectious Diseases, Cluj-Napoca, Romania. We analyzed data from paper and electronic records of adult patients with acute viral infections, including mild to critical forms of COVID-19 and severe influenza, during the 2023–2024 winter season from November 2023 to February 2024. According to the Romanian National Center for Surveillance and Control of Communicable Diseases, the main circulating SARS-CoV-2 variant during this period was Omicron, which was present in multiple subvariant forms, including XBB.1.5, XBB.1.5+F456L, and JN.1 [30]. According to the seasonal influenza 2023–2024 annual epidemiological report made by the European Center for Disease Control, the main circulating influenza virus type was type A, with the main subtypes being A(H1N1)pdm09 and A(H3N2) [31].

### 2.2. Participants and Variables

Inclusion criteria for the study consisted of patients with a confirmed respiratory viral infection, defined as a diagnosis of COVID-19 based on a positive SARS-CoV-2 rapid antigen test or SARS-CoV-2 RT-PCR (GeneXpert-Cepheid, Xpert^®^ Xpress CoV-2/Flu/RSV plus, and DiagCORE-Qiagen, QIAstat-Dx Respiratory SARS-CoV-2 Panel) and a diagnosis of influenza determined by a positive Influenza A/B rapid antigen test or Influenza RT-PCR (GeneXpert-Cepheid, Xpert^®^ Xpress CoV-2/Flu/RSV plus and DiagCORE-Qiagen, QIAstat-Dx Respiratory SARS-CoV-2 Panel), requiring hospitalization. Eligible patients were adults (aged over 18 years) with available laboratory workups, including a complete blood count, biochemical markers, and imaging studies (chest X-ray or CT scan), who consented to the study. Patients with incomplete laboratory workup and hematologic malignancies were excluded.

The data was collected from the hospital’s electronic system, and it included the following: demographics, clinical data, and laboratory tests performed at admission: complete blood count, biochemistry tests, coagulation tests, imaging, ICU admittance, and outcome. Inflammatory markers and comorbidities were collected to provide a comprehensive description of disease status. Severity of the COVID-19 classification was performed at discharge according to the first World Health Organization classification [32,33] and adopted in Romania by a health ministry order on the management of COVID-19 [34]. Patients with severe influenza were defined as those who required hospitalization and oxygen supplementation during their clinical course [35]. This definition is consistent with the criteria used in previous studies, where severe outcomes were associated with the necessity for oxygen therapy and prolonged hospitalization [36,37].

Peripheral blood samples collected within one day of hospital admission were used for the assessment of biomarkers related to vitamin D metabolism, namely, serum vitamin D (25-OH-vitamin D and 1,25-OH-vitamin D) levels and vitamin D receptor single-nucleotide polymorphisms (SNP)—rs1544410, rs731236, and rs7975232. No additional visits for sample collection or monitoring were required from the patients, and informed consent was obtained from each patient.

Patients were treated based on the hospital and national treatment protocol; there was no intervention regarding their treatment and outcome.

The study protocol was approved by the Ethics Committee of “Iuliu Hațieganu” University of Medicine and Pharmacy, Cluj-Napoca, Romania (No. AVZ73/11.03.2022) and by the Infectious Diseases Hospital ethics committee (No. 25868/07.11.2023).

### 2.3. Vitamin D and Polymorphism Receptors Analysis

Plasma levels of 25-OH-vitamin D (code: EQ 6411-9601) were measured using commercially available ELISA kits (EUROIMMUN, 23560 Lübeck, Germany). The procedures followed the manufacturer’s instructions. Absorbance measurements and plate washing were performed with an 800 TS ELISA microplate reader and a Biotek Microplate 50 TS washer (Agilent Technologies, Santa Clara, CA, USA). The interpretation of 25-OH vitamin D plasma levels was based on the American Endocrine Society’s guidelines, with levels classified as deficient (<20 ng/mL), insufficient (21–29 ng/mL), and sufficient (≥30 ng/mL) [38,39].

Genomic DNA was extracted from 200 uL of whole blood using the Invitrogen™ PureLink™ Genomic DNA Mini Kit (catalog number: K182002), following the manufacturer’s protocol. DNA concentration and purity were assessed by checking the 260/280 quality ratio with a spectrophotometer (BioDrop-DUO UV/Vis, Biochrom Ltd., Farnborough, UK). Single-nucleotide polymorphisms (SNP) of vitamin D receptor—rs1544410, rs731236, and rs7975232—were analyzed using the TaqMan SNP Genotyping Assay (catalog number: C___8716062_20, C___2404008_10, and C__28977635_10, respectively) from Thermo Fisher Scientific (Pittsburgh, PA, USA). The amplification and detection of the SNPs were carried out using a QuantStudio 5 Dx Real-Time PCR System (Applied Biosystems, Thermo Fisher Scientific). PCR reactions were set up in a 10 µL final volume containing 2.5 µL of TaqPath ProAmp Master Mix (catalog number: A30866), 0.125 µL of the TaqMan SNP Genotyping Assay, 1 µL of DNA sample, and nuclease-free water, as per the manufacturer’s instructions. Genotyping results were analyzed using the QuantStudio 3/5 Design and Analysis Software.

### 2.4. Statistical Analysis

Descriptive statistics were used to summarize the study population’s baseline and general characteristics, including demographic variables and comorbidities. For the evaluation of normality for the quantitative data, the Shapiro–Wilk test was used. Results were expressed as median with 25th and 75th percentiles. For group comparisons for categorical variables, the chi-squared or Fisher’s exact tests were employed. For comparison of groups, the Kruskal–Wallis one-way ANOVA was used. To assess significant differences among groups, a post hoc pairwise comparison was performed using the Dwass–Steel–Critchlow–Fligner method. We considered *p*-values of <0.05 to be statistically significant. Statistical analysis was performed using Jamovi software, version 2.3 [40,41].

## 3. Results

We included in our study 71 patients who presented to the hospital for COVID-19 or severe influenza during the November 2023 to February 2024 winter season and consented to the study. Of the patients presenting for SARS-CoV-2 virus infection, 26 had a mild form, 22 had moderate COVID-19, and 15 were diagnosed with severe or critical COVID-19. Eight patients presenting with severe influenza were included. Patient demographics, comorbidities, and vaccinal status are presented in Table 1.

Vitamin D deficiency (serum levels ≤20 ng/mL) was identified across all groups in different proportions. Among patients with mild COVID-19, 46.15% (12/26) had vitamin D deficiency, while in the moderate COVID-19 group, this percentage was 36.36% (8/22). Vitamin D deficiency was notably more prevalent in patients with severe COVID-19, affecting 73.33% (11/15). Within the COVID-19 group, while 37 patients had vitamin D levels >20 ng/mL, 11 remained within the insufficient range, and only 26 patients had levels classified as sufficient (>30 ng/dL). Among patients with severe influenza, 37.5% (3/8) had vitamin D deficiency, two remained in the insufficient range, and only three had sufficient vitamin D levels.

In Table 2, we summarize laboratory parameters, including vitamin D levels, inflammatory markers, and biochemical profiles from patients with various severity levels of COVID-19 and severe influenza. Significant differences were observed for Vitamin D levels, neutrophil, lymphocyte, neutrophil-to-lymphocyte ratio (NLR), monocyte counts, CRP, and urea levels.

A post hoc pairwise comparison was performed for the variables with a significant p-value, providing data about significant differences among groups. Severe COVID-19 patients had significantly lower vitamin D levels compared to those with moderate COVID-19 (*p* = 0.014) and influenza (*p* = 0.028). The neutrophil counts were significantly higher in severe COVID-19 patients compared to those with mild (*p* = 0.011) and moderate COVID-19 (*p* = 0.013). Severe COVID-19 patients exhibited significantly lower lymphocyte counts compared to mild (*p* < 0.001) and moderate COVID-19 patients (*p* = 0.004). Patients with severe COVID-19 had significantly higher NLR values compared to those with moderate COVID-19 (*p* = 0.001) and mild COVID-19 (*p* < 0.001). CRP levels were significantly higher in severe COVID-19 patients compared to mild COVID-19 (*p* < 0.001), moderate COVID-19 (*p* = 0.015), and influenza patients (*p* = 0.010). Severe COVID-19 patients had significantly higher urea levels compared to mild COVID-19 (*p* = 0.011) and influenza patients (*p* = 0.037).

For the COVID-19 patients, we performed a comparative statistical analysis between those with vitamin D deficiency (≤20 ng/mL) and those without (>20 ng/mL) in relation to their comorbidities and VDR polymorphisms. The results, summarized in Table 3, indicate that there were no statistically significant differences between the two groups regarding age distribution, sex, or the prevalence of major comorbidities, including cardiovascular, pulmonary, endocrine, and oncological conditions. Similarly, no significant associations were found between vitamin D status and any of the three analyzed VDR polymorphisms (rs1544410, rs731236, and rs7975232).

## 4. Discussion

### 4.1. Summary of Findings

This study aimed to investigate the role of vitamin D levels and vitamin D receptor polymorphisms in the clinical outcomes of patients hospitalized with COVID-19 or severe influenza during the 2023–2024 winter season.

Our findings highlight a strong association between disease severity and inflammatory marker levels, with vitamin D deficiency playing a potential role in modulating these outcomes. In contrast, the distribution of the studied vitamin D receptor gene polymorphisms (rs1544410, rs731236, and rs7975232) did not demonstrate significant associations with either vitamin D status or disease severity.

### 4.2. Vitamin D and Clinical Outcomes in COVID-19

When examining vitamin D concentrations across the three COVID-19 severity classifications, we observed that lower vitamin D levels are associated with disease severity. We also noticed a non-linear pattern: patients with severe disease had the lowest median 25(OH)D levels (8.1 ng/mL; 73.3% deficient), while those with moderate disease showed the highest levels (32.6 ng/mL; 36.4% deficient), and the mild group was slightly lower (20.8 ng/mL; 46.5% deficient). This distribution may be attributed to the fact that mild and moderate cases often share overlapping clinical features and mainly differ by the presence of radiological pneumonia without hypoxemia. For this reason, several studies have collapsed mild and moderate into a single, non-severe group for comparison against severe disease [42,43,44]. However, we retained the three-category classification as it maintains clinical usefulness and avoids a statistical imbalance that would result from a large, non-severe group versus a smaller severe group, which could increase error. For transparency, we also performed a binary comparison (mild/moderate vs. severe), which confirmed significantly lower vitamin D concentrations in severe cases (Mann–Whitney U test, *p* = 0.009).

Overall, our findings are aligned with most of the literature, which reports that lower vitamin D levels are linked to a higher risk of severe illness. For example, in a systematic review and meta-analysis, Pereira et al. pointed out that vitamin D deficiency was associated with severe forms of COVID-19, with patients with vitamin D insufficiency also having increased hospitalization and mortality rates from SARS-CoV-2 infection [45]. Several other studies suggest that low vitamin D levels are associated with increased risk of severe disease and death [46]. A previous study conducted by Topan et al., in the same hospital as our study but during a different period and with an entirely separate patient cohort, reported that vitamin D-deficient patients had a significantly higher prevalence of severe or critical COVID-19 and an increased risk of mortality [43]. In their multivariate analysis, vitamin D deficiency remained an independent predictor of disease severity and death among hospitalized COVID-19 patients.

However, there are also observations on the other side of the spectrum. Hernandez et al. observed that while vitamin D levels were lower in hospitalized COVID-19 patients than in population-based controls, no relationship between vitamin D deficiency and severity of disease was observed [47]. Also, some authors noted that the associations disappeared after adjusting for confounders such as age, BMI, and comorbidities [48,49].

The role of vitamin D supplementation remains under debate. Some studies suggest that vitamin D administration improved clinical outcomes [50,51]. While the pooled data leans towards a potential benefit [14], there is high heterogeneity among studies [13]. Cannata-Andia et al. observed that cholecalciferol supplementation did not improve the outcome of COVID-19, but rather it was the serum vitamin D level at hospital admission that was associated with the outcomes [52].

### 4.3. Vitamin D Receptor Polymorphisms (BsmI, TaqI, and ApaI) in SARS-CoV-2 Infection

In the Romanian population, VDR polymorphism research was primarily focused on dental and endocrine disorders [53,54]. Their possible implications for other health conditions, including infectious diseases such as COVID-19 and influenza, received less attention, and our study seeks to address this gap.

However, before comparing our results with previous studies, it is necessary to address an important issue, which is the inconsistent allele nomenclature used in the VDR literature. The original RFLP (restriction fragment length polymorphism) technique conventionally defined uppercase alleles (A, B, and T) as absence of the restriction site and lowercase alleles (a, b, and t) as presence of the site. However, some recent studies use A/a to denote major and minor alleles, respectively. This can sometimes lead to contradictions, for example, for ApaI (rs7975232), some authors report ‘A = C’ and ‘a = A’, while others invert this mapping. To avoid confusion, we have referred to alleles by their actual nucleotides (e.g., A or C for rs7975232) whenever possible.

#### 4.3.1. rs1544410 (BsmI)

While the evidence on the role of BsmI in COVID-19 is still limited, it leans toward the C allele being a potential risk factor for severity. Al-Gharrawi et al. analyzed whether the rs1544410 polymorphism influences the outcomes of COVID-19 in an Iranian population, by determining it in 1734 and 1450 patients who had recovered or deceased [55]. They found that the CC genotype for BsmI was associated with mortality during the Delta and Omicron waves, with the CT genotype being a risk for the Alpha and Delta variants. Aci et al. reported that the C allele and CC genotype were associated with hospitalization in patients infected with SARS-CoV-2 [56]. Similarly, in an Italian study, the CC genotype showed a higher prevalence in COVID-19 patients compared to healthy individuals [57].

Several studies also examined the relationship between rs1544410 and circulating vitamin D. In the Iranian cohort, low vitamin D levels were associated with the rs1544410 CC genotype, and higher ones with the TT genotype [55]. Aci et al. also noted that patients with the CT and CC genotype had reduced vitamin D levels compared to individuals with the TT genotype [56]. However, other studies report no association between BsmI and serum vitamin D levels [28].

In our study, the distribution of rs1544410 did not significantly differ across disease severity groups or between vitamin D-deficient and non-deficient patients, highlighting the need for research across diverse populations, as most data is available from localized cohorts and might not capture genetic variability worldwide.

#### 4.3.2. rs731236 (TaqI)

The evidence regarding the association of TaqI with COVID-19 outcomes remains mixed and, at times, conflicting. Abdollahzadeh et al. observed no association between TaqI and clinical manifestations or severity [58]. In contrast, a study of a Cuban population reported that the GG genotype was linked to an increased risk of symptomatic and severe forms of COVID-19, although with wide confidence intervals [59]. Another study observed an association between the AA genotype and ICU admission, while the heterozygous AG genotype appeared protective [60].

A very interesting result was observed by Albu-Mohammed et al. They noted that AG and GG genotypes were associated with higher mortality during the Alpha wave, while GG was a risk factor during Delta, but no association was found for Omicron BA.5 [61]. In our cohort with mainly Omicron subvariants, the distribution of rs731236 genotypes also did not differ significantly between severity groups. This highlights the possibility that not only do host genetic backgrounds account for the varied results, but also the genetic characteristics of the virus.

We did not observe any significant TaqI distribution between vitamin D-deficient and non-deficient patients. This is in line with the existing literature, as several other authors also did not identify an association [62,63].

#### 4.3.3. rs7975232 (ApaI)

Research about the ApaI polymorphism in COVID-19 also reveals mixed outcomes. For instance, Jafarpoor et al. found no significant relationship between ApaI polymorphisms and COVID-19 susceptibility [64]. Similarly, Alhamadin et al. reported no significant association between ApaI and disease severity, although they noted that patients with the heterozygous genotype reported fatigue and muscle pain as long-term post-COVID manifestations [29]. In contrast, Abdollahzadeh et al. observed that the heterozygous CA genotype might be associated with a higher risk of severe disease [58], while the AA genotype appeared protective for severe COVID-19 in a small Bangladeshi cohort [65]. On the other hand, Al-Gharrawi et al. observed that patients with the rs7975232 AA genotype had a significantly higher COVID-19 mortality rate [55], highlighting the contradictory data in the published studies.

In regard to vitamin D levels, the CC genotype was associated with the highest serum concentrations, while patients with the AA genotype had the lowest [55].

Our analysis did not reveal significant differences in the distribution of rs7975232 across disease severity groups, nor between patients with and without vitamin D deficiency. These findings suggest that ApaI polymorphisms did not significantly influence acute disease progression or vitamin D levels in our cohort, underscoring the possibility that environmental factors and genetic backgrounds may contribute to the heterogenicity of results reported in the literature.

### 4.4. Vitamin D, Polymorphisms, and Influenza

The role of vitamin D in influenza infection has been studied, with both observational and clinical trials that support its protective effect. A meta-analysis found that vitamin D supplementation significantly reduces the risk of influenza infections [10], with several other studies proving the benefit, especially in pediatric cohorts [66,67]. A study by Hurst et al. evaluated the link between 25(OH)-Vitamin D plasma levels and influenza severity outcomes in 93 patients during the 2009–2010 H1N1 pandemic. They observed a negative association between total 25(OH)D levels and the need for mechanical ventilation, but not with in-hospital mortality [68].

Interestingly, even if most of our influenza patients were in the deficient or insufficient vitamin D categories, our study revealed differences between severe COVID-19 and influenza regarding vitamin D levels. Despite being from the same winter season, the same geographic region, and at a slightly older median age in the influenza group (76.5 years, IQR: 69.5–81.8, vs. 74 years, IQR: 63.5–80), influenza patients exhibited significantly higher vitamin D levels than those with severe COVID-19.

This may point to a possible disease-specific role in vitamin D-related immune modulation, but current evidence is insufficient to confirm such a link. One possible explanation is the relatively shorter duration and acute nature of severe influenza compared to COVID-19, where prolonged inflammation and cytokine production may influence vitamin D levels. However, differences could also be explained by patient comorbidities, concurrent medications, or socio-economic factors. Broader comparative studies are needed to clarify these relationships.

We were unable to identify significant studies addressing the role of BsmI, ApaI, or TaqI in the clinical outcomes of influenza. We, therefore, compared the VDR genotype distributions between severe influenza and COVID-19 cohorts and did not identify any significant difference. This suggests that, at least in our cohort, the polymorphisms do not act as pathogen-specific susceptibility factors. While this comparison must be interpreted with caution due to its small cohort, it contributes to the literature on VDR variants in respiratory infections.

### 4.5. Vitamin D, Immunity, and Inflammation

In our comparison, severe COVID-19 cases were characterized not only by lower vitamin D levels but also by elevated inflammatory markers such as CRP and NLR, which is consistent with previous reports associating these biomarkers with disease severity [69,70]. This may reflect that vitamin D deficiency in severe patients may coexist with heightened systemic inflammation [71,72], though it is possible that they both arise from similar physiological responses or underlying factors.

While we did not identify a causal relationship in our study, there are several potential mechanisms described in the literature that may link vitamin D deficiency to COVID-19 severity. Vitamin D enhances the immune system by promoting the production of antimicrobial peptides, namely cathelicidin (LL-37), and defensins that could help combat respiratory pathogens [73,74], with their antiviral role noted against influenza [75] and RSV [76] in vitro. Some authors note their potential use to prevent severe COVID-19 inflammatory response and reduce the risks of lung injury and thrombosis [77,78]. Also, vitamin D has the ability to decrease the level of proinflammatory cytokines, including IL-6 [79,80], but also to alter nuclear factor-kB activity [81], one of the potential factors responsible for the cytokine storm from the SARS-CoV-2 infection [82].

However, the observed association between low vitamin D levels and severe disease might also reflect reverse causality. Inflammatory processes and acute phase reactions in severe illnesses might decrease circulating vitamin D levels by various mechanisms. Redistribution by promoting the conversion of 25-hydroxyvitamin D to its active form, leading to a reduction in serum levels, and consumption by an increased use of the vitamin in immune regulation during severe inflammation, and by altering the vitamin D binding protein. A solid argument for the redistribution and consumption theory would be the fact that immune cells such as dendritic cells, macrophages, and T lymphocytes express both the vitamin D receptor (VDR) and the enzyme 1α-hydroxylase (CYP27B1), enabling them to locally convert 25-hydroxyvitamin D into its active form, 1,25-dihydroxyvitamin D; however, during severe inflammation, this increased conversion could potentially lead to a decrease in the circulating level of the vitamin [83,84]. Moreover, respiratory epithelial cells have the ability to convert inactive vitamin D into its active form, another possible cause of vitamin redistribution and consumption [85]. Also, in critical illness, factors such as hepatic dysfunction, increased vascular permeability, and fluid shifts can reduce vitamin D concentrations, thereby lowering the total measurable 25(OH)D levels [86,87,88,89]. Reduced vitamin D levels are not only linked with severe/critical forms of COVID-19, but also with other diseases where the underlying cause is an exaggerated immune response, like ARDS [90] and sepsis [91,92,93].

### 4.6. Strengths and Limitations

Existing studies on VDR polymorphisms and COVID-19 severity have yielded mixed results, a variability likely influenced by differences in study populations and by the viral variants circulating at the time of patient recruitment. Our study adds strength to this body of evidence by evaluating a previously under-represented Eastern European cohort during the Omicron subvariant period, when the clinical profile of COVID-19 differed from earlier waves. By adding data from a distinct population and variant context, our study contributes to a more comprehensive understanding of how VDR genetic variation may influence COVID-19 outcomes.

Another key strength of this study is its control over seasonal and regional factors, minimizing potential confounding variables that could affect vitamin D levels and disease severity. The inclusion of both COVID-19 and influenza patients allows for a comparative framework for vitamin D’s potential role in different viral infections, and contributes to the literature on VDR polymorphisms, especially in influenza, where the literature is scarce.

While this study has its strengths, we also need to acknowledge its limitations. The most notable limitation is the relatively small sample size, especially for influenza, which limits the generalization of our findings. Additionally, the cross-sectional design of the study prevents us from assessing pre-infection vitamin D levels and their evolution throughout the disease’s progression. Further studies with larger sample sizes and serial vitamin D level measurements, including a baseline value, would be required to explore the role vitamin D plays in modulating COVID-19 and influenza disease severity.

## 5. Conclusions

Our study analyses the connection between vitamin D levels, inflammatory markers, and disease severity in different forms of COVID-19 and influenza. Our findings highlight a strong association between disease severity and inflammatory marker levels, with vitamin D deficiency playing a potential role in modulating these outcomes. Vitamin D receptor polymorphisms did not show any significant association with vitamin D status and disease severity.

While lower vitamin D levels in severe COVID-19 patients suggest a possible disease-specific modulation, whether this potential association points to causality remains unclear, with studies measuring serial vitamin D levels required to further investigate the process.

Considering our findings and information from the existing literature, routine screening and correction of vitamin D deficiency in high-risk individuals would be the prudent approach, although its direct impact on disease severity warrants further investigation.

## Figures and Tables

**Table 1:** Demographic characteristics, comorbidities, and vaccination status of patients with COVID-19 and severe influenza.

Characteristics	Mild COVID-19	Moderate COVID-19	Severe/Critical COVID-19	Severe Influenza
N	26	22	15	8
Median Age (IQR)	56 (39.3–70)	65.5 (52.3–71.3)	74 (63.5–80)	76.5 (69.5–81.8)
M (%)	11 (42.31)	8 (36.36)	8 (53.33)	4 (50)
F (%)	15 (57.69)	14 (63.64)	7 (46.67)	4 (50)
Cardiovascular (%)	12 (46.15)	14 (63.64)	11 (73.33)	8 (100)
Pulmonary (%)	2 (7.69)	2 (9.09)	1 (6.67)	4 (50)
Diabetes (%)	3 (11.54)	5 (22.73)	7 (46.67)	3 (37.5)
Renal (%)	0	1 (4.55)	3 (20)	2 (25)
Hepatic (%)	4 (15.38)	2 (9.09)	1 (6.67)	0
Endocrine (%)	2 (7.69)	1 (4.55)	4 (26.67)	2 (25)
Neurological (%)	2 (7.69)	0	3 (20)	1 (12.5)
Oncological (%)	1 (3.85)	0	0	0
COVID-19 Vaccination (%)	21 (80.7)	19 (86.3)	5 (33.3)	NA
ICU Admission	0	0	1	0
Mortality	0	0	1	0

Abbreviations: N, number of patients; F, female; M, male.

**Table 2:** Laboratory markers, vitamin D levels, and VDR polymorphisms in COVID-19 and severe influenza.

Parameters		Mild COVID-19 (N = 26)	Moderate COVID-19 (N = 22)	Severe COVID-19 (N = 15)	Severe Influenza (N = 8)	*p*-Value
Vitamin D (ng/mL)		20.8 (7.56–38.7)	32.6 (13.0–38.6)	8.08 (4.79–15.7)	25.6 (18.9–34.5)	**0.022**
Leukocytes (cells/µL)		5900 (5025–7700)	5700 (5258–6375)	8270 (6025–9300)	7440 (5698–9405)	0.086
Neutrophils (cells/µL)		3258 (2432–4612)	3382 (2886–4131)	6390 (4540–7595)	5275 (3095–6250)	**0.006**
Lymphocytes (cells/µL)		1536 (1153–2148)	1417 (1023–1925)	740 (505–1115)	840 (668–1598)	**< ** **0.001**
Monocytes (cells/µL)		723 (547–877)	624 (421–792)	447 (255–550)	405 (278–835)	**0.049**
NLR		1.99 (1.36–3.13)	1.94 (1.71–3.52)	6.6 (4.67–13.2)	7.45 (2.5–12.4)	**<** **0 .001**
Platelets (* 10^3^ cells/µL)		237.5 (184–272.5)	184.5 (142.5–256)	173 (137–254)	182 (163–242)	0.149
CRP (mg/dL)		1.08 (0.515–2.17)	2.11 (1.18–4.93)	8.32 (4.81–13.4)	5.19 (4.57–5.83)	**< ** **0.001**
GOT (U/L)		22.0 (19.3–30.8)	25.5 (22.3–31.8)	35 (24.5–46.5)	33.5 (23.5–50.3)	0.102
GPT (U/L)		25.5 (16.0–31.8)	21.5 (16–33)	26 (16.0–62.5)	27.5 (20.5–33.5)	0.836
Urea (mg/dL)		29.3 (22.5–35.0)	32.3 (26.8–38.0)	39.0 (34.5–94.0)	80.5 (40.0–126)	**0.002**
rs1544410	CC (n, %)	8 (11.3%)	9 (12.7%)	5 (7.0%)	3 (4.2%)	0.921
	CT (n, %)	13 (18.3%)	11 (15.5%)	8 (11.3%)	3 (4.2%)	
	TT (n, %)	5 (7.0%)	2 (2.8%)	2 (2.8%)	2 (2.8%)	
rs731236	AA (n, %)	9 (12.6%)	10 (14.1%)	5 (7%)	4 (5.6%)	0.97
	AG (n, %)	14 (19.7%)	10 (14.1%)	8 (11.2%)	3 (4.2%)	
	GG (n, %)	3 (4.2%)	2 (2.8%)	2 (2.8%)	1 (1.4%)	
rs7975232	CC (n, %)	7 (9.9%)	8 (11.3%)	4 (5.6%)	3 (4.2%)	0.431
	CA (n, %)	9 (12.7%)	10 (14.1%)	9 (12.7%)	2 (2.8%)	
	AA (n, %)	10 (14.1%)	4 (5.6%)	2 (2.8%)	3 (4.2%)	

Abbreviations: NLR, neutrophil-to-lymphocyte ratio; CRP, C-reactive protein; GOT, glutamic oxaloacetic transaminase; GPT, glutamate pyruvate transaminase.

**Table 3:** Comorbidities and VDR polymorphisms in COVID-19 patients by vitamin D status.

Parameters		Vitamin D ≤ 20	Vitamin D > 20	*p*-Value
N		34	37	-
Age ≥ 65 (%)		18(52.94%)	19(51.35%)	0.893
M (%)		16 (47.06%)	15 (40.54%)	0.58
F (%)		18 (52.94%)	22 (59.46%)	
Cardiovascular (%)		19 (55.88%)	26 (70.27%)	0.209
Pulmonary (%)		3 (8.82%)	6 (16.22%)	0.482 *
Diabetes (%)		11 (32.35%)	7 (18.92%)	0.194
Renal (%)		4 (11.76%)	2 (5.41%)	0.417 *
Hepatic (%)		4 (11.76%)	3 (8.11%)	0.703 *
Endocrine (%)		4 (11.76%)	5 (13.51%)	1 *
Neurological (%)		3 (8.82%)	3 (8.11%)	1 *
Oncological (%)		1 (2.94%)	0 (0.00%)	1 *
rs1544410	CC (n, %)	13 (38.2%)	12 (32.4%)	0.683
	CT (n, %)	17 (50.0%)	18 (48.6%)	
	TT (n, %)	4 (11.8%)	7 (18.9%)	
rs731236	AA (n, %)	15 (44.1%)	13 (35.1%)	0.679
	AG (n, %)	16 (47.1%)	19 (51.3%)	
	GG (n, %)	3 (8.8%)	5 (13.5%)	
rs7975232	CC (n, %)	12 (35.3%)	10 (27.0%)	0.725
	CA (n, %)	13 (38.2%)	17 (45.9%)	
	AA (n, %)	9 (26.5%)	10 (27.0%)	

* Fisher’s exact test.

## Data Availability

The datasets presented in this article are not readily available because the data are part of an ongoing study.

## Abbreviations

The following abbreviations are used in this manuscript:

ARDS	Acute respiratory distress syndrome
A	Adenine
COVID-19	Coronavirus disease, 2019
C	Cytosine
CRP	C-reactive protein
CT	Computed tomography
DNA	Deoxyribonucleic acid
ELISA	Enzyme-linked immunosorbent assay
G	Guanine
ICU	Intensive care unit
IQR	Interquartile range
NLR	Neutrophil to lymphocyte ratio
NG/mL	Nanograms per milliliter
NA	Not applicable
PCR	Polymerase chain reaction
RT-PCT	Polymerase chain reaction
SARS-CoV-2	Severe acute respiratory syndrome coronavirus 2
SNP	Single-nucleotide polymorphism
T	Thymine
VDR	Vitamin D receptor
WHO	World Health Organization
